# Alterations in the *Aedes aegypti* Transcriptome during Infection with West Nile, Dengue and Yellow Fever Viruses

**DOI:** 10.1371/journal.ppat.1002189

**Published:** 2011-09-01

**Authors:** Tonya M. Colpitts, Jonathan Cox, Dana L. Vanlandingham, Fabiana M. Feitosa, Gong Cheng, Sebastian Kurscheid, Penghua Wang, Manoj N. Krishnan, Stephen Higgs, Erol Fikrig

**Affiliations:** 1 Section of Infectious Diseases, Department of Internal Medicine, Yale University School of Medicine, New Haven, Connecticut, United States of America; 2 Department of Pathology, University of Texas Medical Branch, Galveston, Texas, United States of America; 3 Howard Hughes Medical Institute, Chevy Chase, Maryland, United States of America; The Rockefeller University, United States of America

## Abstract

West Nile (WNV), dengue (DENV) and yellow fever (YFV) viruses are (re)emerging, mosquito-borne flaviviruses that cause human disease and mortality worldwide. Alterations in mosquito gene expression common and unique to individual flaviviral infections are poorly understood. Here, we present a microarray analysis of the *Aedes aegypti* transcriptome over time during infection with DENV, WNV or YFV. We identified 203 mosquito genes that were ≥5-fold differentially up-regulated (DUR) and 202 genes that were ≥10-fold differentially down-regulated (DDR) during infection with one of the three flaviviruses. Comparative analysis revealed that the expression profile of 20 DUR genes and 15 DDR genes was quite similar between the three flaviviruses on D1 of infection, indicating a potentially conserved transcriptomic signature of flaviviral infection. Bioinformatics analysis revealed changes in expression of genes from diverse cellular processes, including ion binding, transport, metabolic processes and peptidase activity. We also demonstrate that virally-regulated gene expression is tissue-specific. The overexpression of several virally down-regulated genes decreased WNV infection in mosquito cells and *Aedes aegypti* mosquitoes. Among these, a pupal cuticle protein was shown to bind WNV envelope protein, leading to inhibition of infection *in vitro* and the prevention of lethal WNV encephalitis in mice. This work provides an extensive list of targets for controlling flaviviral infection in mosquitoes that may also be used to develop broad preventative and therapeutic measures for multiple flaviviruses.

## Author Summary

Dengue (DENV), West Nile (WNV) and Yellow Fever (YFV) viruses are responsible for severe human disease and mortality worldwide. There is no vaccine available for dengue or West Nile virus and no specific antiviral is available for any of these viral infections. These viruses are transmitted to humans through the bite of a mosquito vector. Understanding the effects of viral infection on gene expression in the mosquito is crucial to the development of effective antiviral treatments for mosquitoes and may enable researchers to interrupt the human-insect infection cycle. Here we investigate the alterations in gene expression across the entire *Aedes aegypti* genome during infection with DENV, YFV and WNV over time. We describe several genes that share a similar expression profile during infection with all three viruses. We also use a WNV mosquito cell, mosquito and mouse model to show that virally downregulated genes are inhibitory to infection when overexpressed and that viral regulation of mosquito genes is tissue-specific. Our results provide an extensive amount of data highlighting viral gene targets in the mosquito during infection. This data may also be used to develop broad-spectrum anti-flaviviral treatments in mosquitoes.

## Introduction

West Nile (WNV), dengue (DENV) and yellow fever (YFV) viruses are globally important, re-emerging mosquito-borne flaviviruses that cause widespread human disease and mortality [1]. WNV can cause serious illness in man, resulting in encephalitis and death, and is soon expected to be endemic in most of the United States and South America [1], [2]. DENV is among the most important human infectious diseases globally. There are an estimated 100 million cases per year, with over 500,000 cases of potentially fatal dengue hemorrhagic fever [3], [4]. There is no specific treatment for either West Nile or dengue virus, and efforts to create an effective dengue vaccine have been hindered due to safety concerns and potential antibody-dependent enhancement [3], [5]. YFV is endemic to tropical regions of Africa and South America and causes a febrile illness often involving hemorrhagic manifestations with fatality rates up to 50% [6], [7]. There is a YFV vaccine available but it is underutilized in many countries with endemic YFV and no specific antiviral is available [8].

Flaviviruses typically replicate within a mosquito vector for 7–10 days before the vector can transmit virus to humans [1], [5]. Several recent studies have profiled gene expression during the course of flavivirus infection in mosquitoes [9], [10], [11], [12], [13], [14], [15], [16], [17]. Innate immune genes are the focus of many of these investigations, and the Toll, Janus kinase (JAK)-signal transducer and activator of transcription (STAT) pathways have emerged as important anti-flaviviral mechanisms in the mosquito [9], [10]. There is also evidence that DENV actively suppresses mosquito immune responses *in vitro* [18]. Serine proteases have been shown to be important for both blood digestion and viral propagation, though it is not clear whether they aid or impair viral infection in the mosquito [14], [19]. An RNA interference screen in Drosophila melanogaster cells identified many DENV insect host factors that were shown to be relevant for human cells as well as mosquitoes *in vivo* [11]. In addition, a recent transcriptomic analysis of *Culex quinquefasciatus* genes revealed many common and distinct pathogen-response genes to infection with WNV, *Wuchereria bancrofti* and non-native bacteria, including many genes involved in metabolism and transport [12].

Previous studies investigating gene expression in response to viral infection in both insect and human cells have primarily focused on one flavivirus and/or one gene family. Further investigation is necessary for a global picture of the impact on mosquito gene expression throughout the course of infection with individual flaviviruses. Here, we use comprehensive microarray analysis to identify key alterations in the *Ae. aegypti* transcriptome during infection with WNV, DENV or YFV. The *Ae. aegypti* mosquito is a major vector for numerous flaviviruses [20] and is an ideal model for viral infection studies since the genome has been sequenced and characterized [21]. In addition, *Ae. aegypti* are susceptible to flaviviral infection with WNV in the laboratory and pose a threat for WNV transmission in nature [22], [23]. The flaviviruses used in this study were chosen based on global prevalence and to represent both encephalitic and non-encephalitic flavivirus clades [24]. Analysis was performed on day 1, day 2 and day 7 (D1, D2 and D7, respectively) to observe changes in gene expression at both early and late stages of infection. We report on 405 differentially expressed genes during infection with the three flaviviruses over the three timepoints. This is the first study to our knowledge that compares and contrasts mosquito gene expression in response to multiple flavivirus infection over time.

## Results

### Identification of transcripts altered by flavivirus infection

**Figure 1:**
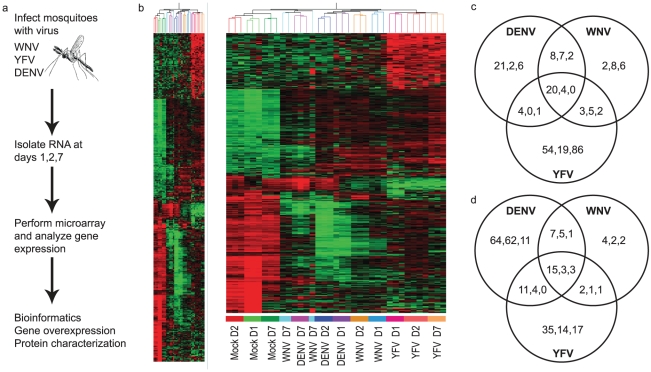
Genome-wide microarray analysis of the mosquito transcriptome during WNV, DENV and YFV infection. A. Schematic of the experimental procedure. *Ae.aegypti* mosquitoes were infected with WNV, DENV or YFV. Microarray analysis was done at 1, 2 and 7 days p.i. for 15,959 genes. B. Heatmap for mosquito genes that were ≥5-fold up-regulated (203 genes) and ≥10-fold down-regulated (202 genes) during infection with any virus at any timepoint. Red, black and green colors indicate gene expression above, equal to and below the mean, respectively. C,D. Venn diagrams of the ≥5-fold up- (C) and ≥10-fold down-regulated (D) genes common and unique to each virus. Timepoints are indicated as D1/D2/D7 within each grouping. The complete dataset can be found in Table S1.

**Figure 2:**
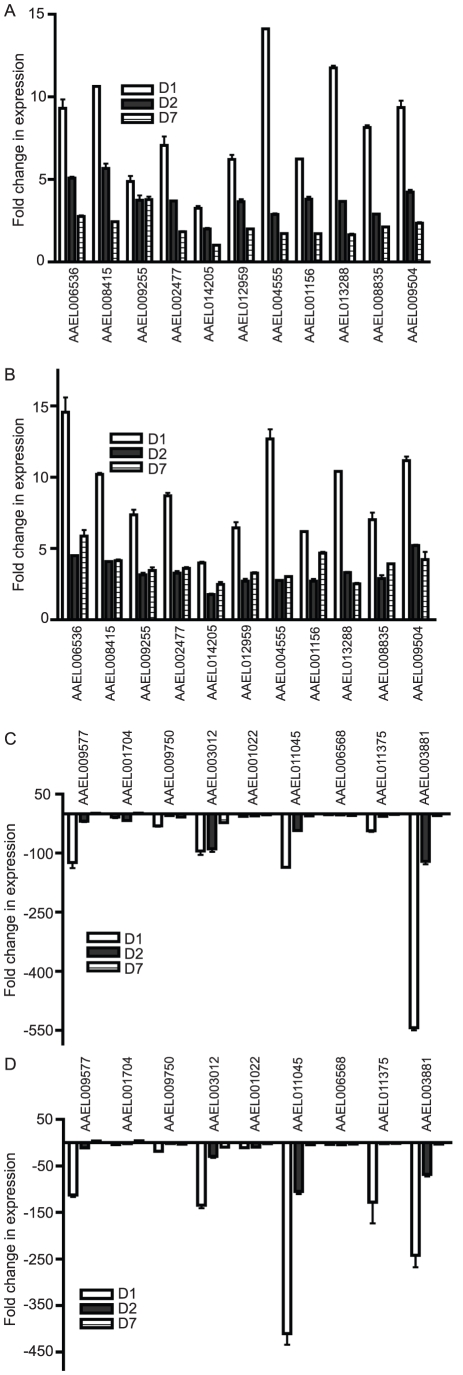
Expression of individual *Ae. aegypti* DEGs at 4 timepoints after WNV or DENV infection. Mosquitoes were infected with virus via blood feeding and RNA isolated at 1, 2, 7 and 14 days post-infection. qRT-PCR analysis was done to measure expression of DURGs (A,B) and DDRGs (C,D) after WNV (A,C) or DENV (B,D) infection. Data from 3 separate infections were analyzed in triplicate and plotted on graphs, error bars indicate standard deviation. Fold change in expression was calculated from Ct values, normalized to actin and compared to mock infection.

We used an established protocol for mosquito inoculation to infect the Rockefeller strain of *Aedes aegypti* mosquitoes with one of three flaviviruses, WNV, DENV or YFV, or mock solution via intrathoracic injection [13]. This method was chosen to positively infect the mosquitoes as well as ensure an even distribution of virus between individual mosquito groups. On D1, D2 and D7, RNA was isolated from the mosquitoes and subjected to genome-wide microarray analysis to determine alterations in gene expression between the mock and infected groups. Analysis was done using a custom X4 NimbleGen array designed against the published *Aedes aegypti* genome [21]. The experiment schematic is shown in Figure 1A. The complete microarray data set can be found in Table S1 and at the NCBI Gene Expression Omnibus (GEO) #GSE28208. Cut-offs of ≥5-fold or ≥10-fold were applied to identify either differentially up-regulated genes (DURGs) or differentially down-regulated genes (DDRGs), respectively. We identified 405 differentially expressed genes (DEGs) during infection with the three flaviviruses. The highest number of DEGs was observed on D1 of infection with all three viruses and the fewest DEGs were found on D7 (Figure S1). DURG and DDRG expression was confirmed using quantitative (q)RT-PCR, analyzing the expression of 11 highly DURGs and 9 highly DDRGs on D1, D2, D7 and D14 in a separate group of *Ae. aegypti* mosquitoes infected with WNV and DENV (Figure 2). Table 1 lists these genes with predicted function in the mosquito as well as any homolog identified through a BLAST search using the *Drosophila melanogaster* genome. Overall, the extent of downregulation of gene transcripts was more dramatic than the upregulation during infection with these flaviviruses. The DDRGs analyzed by (q)RT-PCR showed up to 500-fold decrease in expression during virus infection compared to controls, likely due to the sensitivity of this method of analysis.

**Table 1:** Predicted *Ae. aegypti* gene function and closest homolog in *Drosophila*.

*Ae.aegypti* ****SEQ ID****	*Drosophila* ****ID****	*Drosophila* ****gene name****	Predicted function in mosquito
AAEL009577	CG17052	Obstructor A	Chitin binding, peritrophic membrane
AAEL001704	CG13042		
AAEL009750	None		
AAEL003012	CG1009	Puromycin-sensitive aminopeptidase	Zinc metalloprotease
AAEL001022	CG1803	Regucalcin, isoform D	Organism reproduction
AAEL011045	CG13935	cuticular protein 62Bb, isoform A	Cuticle structure
AAEL006568	CG16705	Spatzle-Processing enzyme	Serine endopeptidase activity, innate immune response, Toll positive regulation
AAEL011375	CG7996	Snake, isoform A	Structural constituent of ribosome, translation, protein modification
AAEL003881	CG2960	Ribosomal protein L40	Structural constituent of ribosome, translation, protein modification
AAEL006536	CG34453		
AAEL008415	CG31301		Nucleic acid binding
AAEL009255	None		
AAEL002477	CG15479		
AAEL014205	None		
AAEL012959	None		
AAEL004555	None		
AAEL001156	CG5280		
AAEL013288	CG7523		
AAEL008835	None		
AAEL009504	None		

### Clustering of altered transcripts

**Figure 3:**
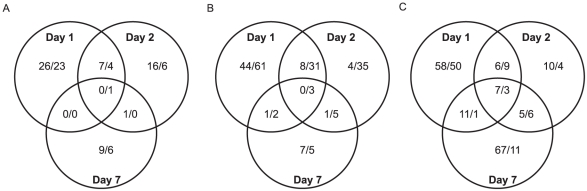
Venn diagrams. Venn diagrams of the ≥5-fold up- and ≥10-fold down-regulated genes common between timepoints for infection with WNV (A), DENV (B) and YFV (C). Up- and down-regulated genes are indicated as up/down within each grouping.

For a global perspective on gene regulation during flaviviral infection, hierarchical clustering analysis was done for all DURGs and DDRGs using the Pearson correlation of gene expressions to generate a heatmap (Figure 1B). Detailed heatmaps indicating levels of individual genes can be found in Figures S2A-G. Extensive clustering was found for the three timepoints during YFV infection, on D7 for WNV and DENV infection, and between D1 and D2 for DENV infection. Significant clustering was also evident for all flaviviruses at all timepoints versus mock infection. These results highlight both similarities and differences in gene regulation by individual flaviviral infections. Further analysis revealed 20 DURGs in common between the three viruses on D1 of infection (Figure 1C), including AAEL014440, a putative juvenile hormone-inducible protein. There were only four DURGs in common on D2 of infection with all three flaviviruses, including AAEL004861-RA, a peroxisomal integral membrane protein (Per8p) and three hypothetical proteins. WNV and DENV shared seven DURGs on D2 of infection, all hypothetical conserved proteins. There were no DURGs in common on D7 of infection with all three flaviviruses. YFV had the most unique DURGs, with 54 on D1, 19 on D2 and 86 on D7. We also noted common and unique DDRGs during infection (Figure 1D), with 15 shared between the three infections on D1, including AAEL011045, a pupal cuticle (PC) protein, and AAEL003012, a matrix metalloprotease (MMP), which were significantly down-regulated. The most unique DDRGs were found during DENV infection, with 64 on D1, including AAEL012402, which codes for an elongase protein, and 62 on D2, including AAEL014108, which codes for an aquaporin protein. DENV and YFV shared 11 DDRGs on D1 of infection, the most between any two flaviviruses at any timepoint, including AAEL004897, a brain chitinase, and two cytochrome P450 genes, AAEL012770 and AAEL000340. Looking at overlapping DEGs throughout infection with any given virus, we found considerable variation between timepoints (Figure 3). During DENV infection, no genes were differentially expressed at all timepoints, while YFV had seven DURGs and three DDRGs in common during infection at D1, D2 and D7. There were slightly more DEGs in common between D1 and D2 for each infection, with 8 DURGs and 31 DDRGs shared on D1 and D2 during infection with DENV (Figure 3).

### Functional annotations of differentially expressed transcripts

**Figure 4:**
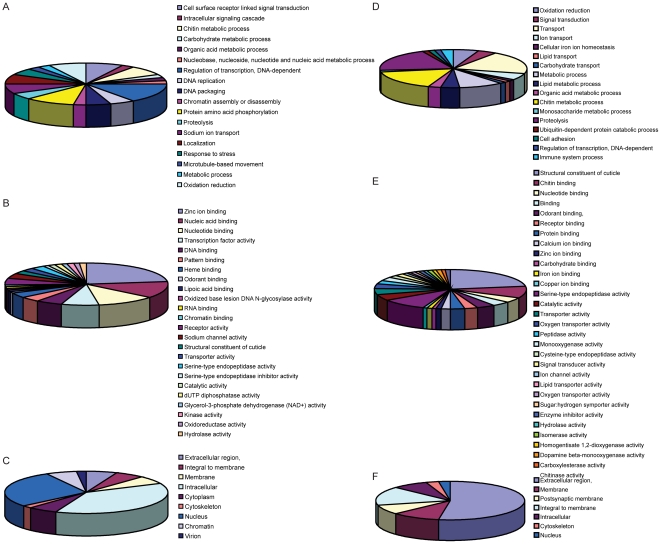
Functional annotations of DEGs. DURGs (A–C) and DDRGs (D–F) were assigned to functional categories for biological process (A,D), molecular function (B,E) and cellular location (C,F). Genes with no known annotation are not included in the pie charts. The full list of annotations can be found in Table S2.

**Figure 5:**
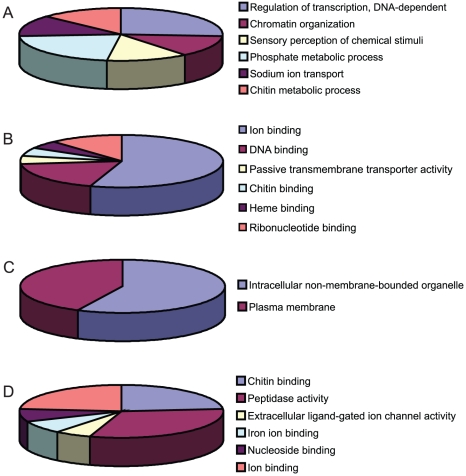
Functional clustering of DEGs. DURGs (A–C) and DDRGs (D) were assigned to functional categories for biological process (A), molecular function (B,D) and cellular component (C). Genes with no known annotation are not included in the pie charts. The full list of functional clustering annotations can be found in Table S3.

The 405 genes that were differentially expressed during infection with one of the flaviviruses were classified into groups based on biological process (BP), molecular function (MF) and cellular component (CC) (Figure 4, Tables S2 and S3.) Genes that did not have any annotation were excluded from analysis. The largest proportions of DURGs with BP annotation were involved in DNA-dependent transcription regulation (3%) and protein amino acid phosphorylation (2%) (Figure 4A). The most abundant MFs among DURGs were zinc ion binding (9%), nucleic acid binding (5%), nucleotide binding (4%) and transcription factor binding (3%) (Figure 4B). The most abundant BP of the DDRGs were associated with chitin metabolism (12%), transport (7%) and proteolysis (7%) (Figure 3D) and the MFs were related to structural constituent of cuticle (23%) and serine-type endopeptidase activity (10%) (Figure 3E). A similar reduction in chitin-binding proteins and alteration in transport genes was found during infection of *Aedes* with Sindbis virus [25]. In addition, metabolism was previously found to be altered by DENV infection on day 10 post-infection [10]. Most of the DURGs were found to be intracellular (9%) and nuclear (7%) (Figure 4C) and most of the DDRGs were extracellular (10%) (Figure 4F). Functional clustering of DEGs revealed that the most significant BPs of DURGs were regulation of transcription (26%) and phosphate metabolic process (22%) and MF clustered at ion binding (55%) (Figure 5A and 5B.) The clustering of DURGs placed most of them in intracellular non-membrane-bound organelles at a surprisingly high 57%, with 43% at the plasma membrane (Figure 5C). For the DDRGs, only MF had significant functional clustering, with the majority involved in peptidase activity (31%), chitin binding (24%) and ion binding (24%).

### Virally-downregulated gene expression is tissue-specific

**Figure 6:**
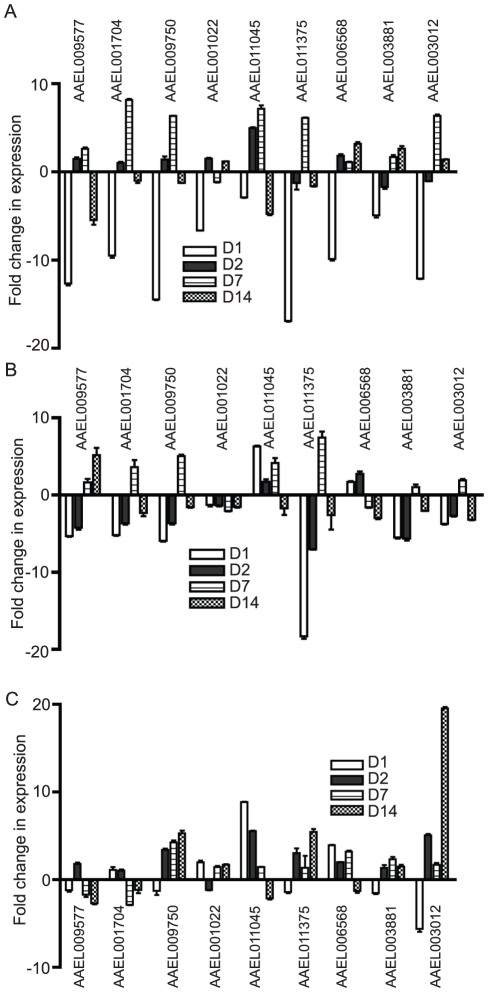
Alteration in DDRG expression in *Ae. aegypti* is tissue-specific. Gene expression was analyzed by qRT-PCR in mosquito tissues at select timepoints during WNV infection of *Ae. aegypti* mosquitoes. The fold change in expression of indicated genes in midgut (A), salivary gland (B) and abdomen (C), after WNV infection compared to mock infection. D1, D2, D7, D14 indicated by representative bars. Data is from 3 separate infection groups and qRT-PCR was done in triplicate. Error bars indicate standard deviation.

During infection of the mosquito, flaviviruses must disseminate from the midgut (MG) through the body to the salivary gland (SG) and so likely alter gene expression in different organs at various times. To determine tissue-specific expression of the identified DEGs, we infected *Ae. aegypti* with WNV through blood feeding and dissected the MG, abdomen (AB) and SG on D1, D2, D7 and D14 p.i.. We performed qRT-PCR on select DDRGs at each timepoint to determine levels of gene expression in each tissue (Figure 6). On D1, all DDRGs tested were significantly downregulated in the MG, which is expected as this is where the virus localizes immediately after feeding. Surprisingly, AAEL011375 was also differentially downregulated in the SG on D1 and D2 p.i. when the virus is not expected to be present. It is possible that signalling molecules travel from the infected MG throughout the mosquito early in infection, affecting gene expression in other organs. By D7 p.i., many DDRGs are upregulated in the MG, possibly to compensate for previous downregulation. At D14 p.i., most DDRGs are downregulated in the SG, which may be indicative of the virus disseminating to this organ by this timepoint. An exception to this is AAEL009577, which is slightly upregulated in the SG and downregulated in the MG at D14 p.i. This could be due to a precise role the protein plays in the mosquito during infection that is not related to viral dissemination. The putative MMP (AAEL003012), found to be highly downregulated in the whole mosquito during infection with all three flaviviruses at all timepoints, was significantly underexpressed in the MG on D1 (12-fold) and then highly upregulated in the AB by D14 (19.5-fold) p.i. In fact, most of the genes that were highly downregulated in the whole mosquito during infection with all three flaviviruses are not significantly downregulated, and often slightly upregulated, in the AB at most timepoints tested. These results suggest a complex balance of gene regulation that occurs in the various organs during flaviviral infection.

### Virally-downregulated genes inhibit WNV infection in mosquito cells and live mosquitoes

**Figure 7:**
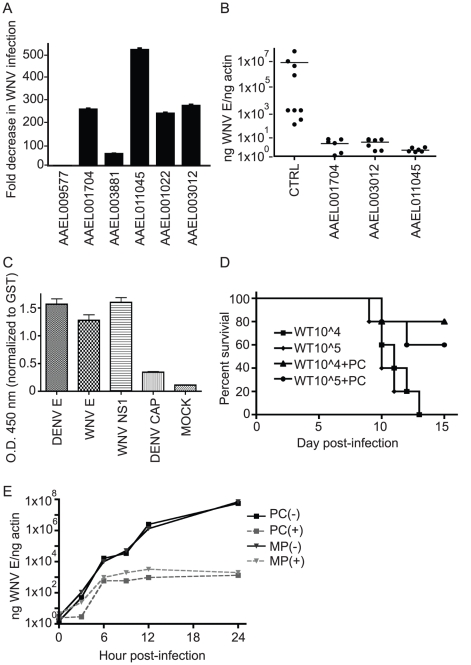
Overexpression of virally down-regulated genes impairs WNV infectivity. A. Overexpression of DDRGs reduces WNV infection of *Ae. aegypti* mosquito cells. CCL-125 cells were transfected with expression plasmids encoding mosquito DDRGs and infected with WNV 48 hours post-transfection (p.t.). Cells were analyzed for infection by immunofluorescence microscopy 24 hours post-infection (p.i.) using an antibody against WNV envelope protein. Fold decrease in infection is indicated. Data is pooled from 3 separate experiments, error bars indicate standard deviation. B. Overexpression of DDRGs reduces WNV infection of *Ae. aegypti* mosquitoes. Mosquitoes were injected with plasmids used in (A) and infected with WNV 6 days p.t. At 10 days p.i., RNA was isolated and qRT-PCR performed to detect WNV. Infection is indicated as ng WNV E/ng actin. p<.001 for all 3 genes vs. control, one way ANOVA and Kruskal-Wallis test were used, n = 10. C. ELISA analysis of AAEL011045 (PC protein) binding to WNV E, DENV E, WNV NS1 and DENV capsid. Optical density (O.D.) is shown at 450 nm and normalized to glutathione S-transferase (GST) binding. Data is pooled from 3 separate experiments, error bars indicate standard deviation. D. Purified recombinant pupal cuticle protein (PC) (AAEL011045) was incubated with WNV for an hour at 37°C and injected i.p. into c57L/B6 mice. Survival curve was plotted for: 10^4^ WNV alone, 10^5^ WNV alone, 10^4^ WNV+PC, 10^5^ WNV+PC; number refers to viral titer in plaque forming units (pfu). p<.05 using the Logrank test. Results are pooled from 2 separate experiments, total n = 10 for each group. E. Overexpression of PC and MP protein affects WNV infection in mosquito cells. CCL-125 cells were transfected as in (A) and analyzed by qRT-PCR as in (B). Points plotted on line graph are from 3,6,9,12 and 24 h post-infection.

Six highly DDRGs from different functional groups that shared similar expression profiles during all three flaviviral infections were selected for further characterization, testing the notion that these genes represent a conserved principal in flaviviral infection. We chose to use WNV to characterize these genes as both mosquito cells and live mosquitoes are highly susceptible to infection and a WNV mouse model of infection and disease has been well-established, with infected mice developing encephalitis that leads to death [26]. We hypothesized that virally down-regulated genes may be inhibitory to infection. First, the genes were expressed in *Ae. aegypti* cells and the susceptibility of the cells to WNV infection was examined using immunofluorescence microscopy with antibodies against WNV envelope protein. A representative image of WNV-infected cells can be found in Figure S3. The overexpression of four previously identified virally-DDRG genes, AAEL001704, AAEL011045, AAEL001022 and AAEL003012, caused a significant reduction in WNV infection of CCL-125 mosquito cells (266.8+/−5.1, 548.75+/−7, 247.28+/−5.9, 284.05+/−6, fold reduction, respectively) compared to GFP alone (Figure 7A). These genes were also shown to inhibit DENV infection of mosquito cells (data not shown). Next, the effect of these proteins on mosquito infection *in vivo* was investigated. It was previously reported that whole body transfection (WBT) of DNA plasmids into mosquitoes results in high expression of target genes [27]. This method was improved by adding a lipid-based transfection reagent to the protocol, which enhanced expression of transfected genes on D3 and D14 post-WBT (p.WBT) (Figure S4). *Ae. aegypti* were transfected by intra-thoracic inoculation with DNA plasmids encoding either GFP, AAEL001704, AAEL011045, or AAEL003012 and infected with WNV through blood feeding 6 days p.WBT. At day 10 p.i., the level of WNV in the mosquitoes was measured using qRT-PCR. The expression of each of the three genes significantly lowered WNV infection by approximately one million-fold (Figure 7B). These results strongly support the hypothesis that these proteins play an important role during virus infection of the mosquito.

### Virally-downregulated pupal cuticle protein inhibits WNV infection in mice by binding to envelope protein and inhibiting viral entry

Since the expression of the selected DDRGs lowered infection both *in vitro* and *in vivo*, we hypothesized that interaction of the mosquito proteins with WNV may be responsible for the inhibition. Recombinant protein was generated from two of the DDRGs that highly inhibited infection: AAEL011045, which codes for a pupal cuticle (PC) protein, and AAEL003012, which produces a putative matrix metalloprotease (MMP). We first investigated if the mosquito proteins interact with viral proteins using ELISA assays to detect potential binding of PC and MMP with DENV envelope (E), WNV envelope (E), DENV capsid (C) and WNV NS1 proteins. PC protein bound DENV E, WNV E and WNV NS1 but the MMP protein did not bind any viral protein (Figure 7C). Incubation of PC or MMP with each of the four viral proteins for one hour at 37°C, followed by Western blot analysis using antibodies against the viral proteins, showed altered protein profiles (Figure S5.) This indicates that PC directly binds viral proteins and that both PC and MMP contribute to increased oligomerization of WNV E, DENV E and WNV NS1. Since PC bound WNV E protein, which is found on the surface of the virion and is responsible for receptor-binding and virus entry, it suggested that the interaction may potentially inhibit WNV cell entry and subsequent infection. To investigate this, we evaluated the effects of both PC and MMP protein interactions with WNV on infection *in vivo* in mice. MMP or PC was incubated with WNV for an hour at 37°C and the solution was injected into mice intraperitoneally (i.p.). Mice that received WNV pre-incubated with PC protein had significantly higher survival rates, with approximately 60% surviving infection, than mice that received either 10^4^ or 10^5^ pfu of WNV, all of which died within 14 days p.i. (Figure 7D). None of the mice that received virus pre-incubated with MMP protein survived WNV infection, even though increased levels of both proteins reduced infection of mosquito cells and live *Ae. aegypti*. This indicates that PC and MMP likely use different mechanisms to inhibit infection in mosquitoes. The protein binding data, the *in vitro* WNV infection data, and the *in vivo* WNV mosquito and mouse infection data together suggested that the mosquito PC protein binds the E protein on the flavivirus surface to directly decrease the potential for infection.

To further investigate the mechanism that the PC protein uses to inhibit viral infection, we looked at the effects of both MMP and PC expression on WNV infection over time in CCL-125 *Ae. aegypti* mosquito cells. Insect expression plasmids encoding each protein were transfected into mosquito cells along with a plasmid expressing green fluorescent protein. The cells were sorted and infected with WNV. The expression of both proteins impaired the ability of WNV to infect the cells (Figure 7E), though at different times. At 3 hours p.i,, the expression of PC resulted in over ten-fold lower levels of viral RNA than the expression of MMP or in cells with no protein overexpression. The level of viral RNA with PC overexpression was below 10 at 3 hours p.i., while the level with MMP or with no increased protein is approximately 10^2^. This indicates that PC likely affects viral entry and that MMP may not be interfering with this step of infection. By 6 hours p.i, both MMP and PC protein expression resulted in an almost 2-log difference in viral RNA levels when compared to cells with no protein expression. By 24 hours p.i., the cells expressing either MMP or PC were significantly less infected than the control cells, with an almost 4-log difference in viral RNA levels. These data agree with our findings that the MMP protein reduced WNV infection in both mosquito cells and mosquitoes *in vivo* yet is unable to directly inhibit WNV in solution from infecting mice, while the PC protein was able to prevent WNV from causing significant infection in each of those scenarios. This further indicates that PC may be inhibiting infection from the point of entry by binding to the E protein while MMP may indirectly inhibit infection at a later point.

## Discussion

Discovery of host factors regulated during viral infection of the mosquito may identify conserved protein families and pathways representing both mosquito anti-viral mechanisms as well as requirements for viral life cycles. Our analysis highlights many mosquito genes that are important for infection with three globally (re)emerging flaviviruses, West Nile, dengue and yellow fever. In our studies, we used *Aedes aegypti* as an *in vivo* model of flavivirus infection. Though the *Ae. aegypti* mosquito is a major vector for DENV and YFV transmission in nature, it is generally considered a secondary vector for the transmission of WNV. The major vector for WNV transmission is the *Culex* mosquito, though WNV has been detected in both *Ae. aegypti* and *Ae. albopictus* in nature [28], [29]. In addition, both *Aedes* and *Culex* mosquitoes are members of the Culicinae subfamily of mosquito vectors. Comparative analysis revealed that the expression profile of 20 significantly upregulated genes and 15 downregulated genes is quite similar between the three flaviviruses on D1 of infection, indicating a potentially conserved transcriptomic signature of flaviviral infection. Indeed, while *Aedes* is the major vector for both DENV and YFV and a secondary vector for WNV, we found a similar overlap of gene expression profiles between WNV and DENV as we found for YFV and DENV. This suggests that there are flavivirus-specific alterations in the mosquito transcriptome regardless of which flavivirus infects the mosquito as well as which mosquito is the major vector of the flavivirus used.

One of the genes significantly upregulated during all three infections was juvenile hormone-inducible protein (AAEL014440), which has a homolog in *Drosophila melanogaster* that is thought to regulate the expression of many other genes [30]. Another gene, core histone H3 (AAEL003685), was over 4-fold upregulated at all timepoints during infection with all three viruses. Several viral proteins target host chromatin and histone proteins to interfere with host gene expression and nucleosome assembly by various mechanisms and for diverse purposes [31]. Herpes simplex virus type 1 (HSV-1) is known to utilize histones for its own genome during lytic infection [32]. The importance of histone proteins in flaviviral infection, and infection in the mosquito vector in general, remains to be investigated. Many genes were found to be differentially regulated in mosquitoes infected with two of the three flaviviruses. For example, during infection with both DENV and WNV, AAEL009750 was over 20-fold downregulated on D1 of infection and significantly lower than mock infection at other timepoints but expression was not significantly altered during YFV infection. This gene codes for a member of the mosquito allergen proteins, which are known to bind human IgE [33] and could interfere with viral transmission to humans if produced in abundance during infection. An interesting point is whether flaviviral infection is altering gene expression directly or indirectly, for example through the enrichment of small interfering RNA molecules. To investigate this, we compared highly downregulated genes from our DENV infected mosquitoes to the recent publication by Hess *et al* regarding small RNA levels during DENV infection and found several correlations [34]. For example, AAEL010160 was downregulated 18.8, 59.3 and 7.45-fold on days 1, 2 and 7 of DENV infection in our analysis, respectively, and a corresponding sense sRNA was enriched with 2.15 log fold-change on day 2 of DENV infection from the dataset published by Hess *et al*. Another gene, AAEL001953, was downregulated 11.84, 19.05 and 3.84-fold on days 1, 2 and 7 during DENV infection in our analysis, respectively, and a corresponding sense sRNA was enriched with 3.65 log fold-change on day 2 of DENV infection in the previous study [34]. This indicates that flaviviruses likely alter gene expression through both direct and indirect mechanisms during infection of the mosquito.

The majority of highly altered mosquito transcripts were not canonical innate immune genes though we were able to correlate our analysis with previous data on viral infection and mosquito immunity. Previous studies suggest that depleting PIAS (protein inhibitor of activated STAT), a negative regulator of the Jak-STAT pathway, resulted in down-regulation of five antimicrobial genes (four Cecropin A-like genes and one defensin l-like gene) that were also downregulated by DENV infection [9]. All five of these genes were significantly downregulated in our analysis during infection with all three flaviviruses infections at all three timepoints. One of these genes, AAEL000611, was highly downregulated late in infection, with 15-fold, 27-fold and 38-fold lower expression on D7 of YFV, DENV and WNV, respectively. Another Cecropin A-like gene, AAEL000627, was also highly downregulated on D7 of infection, with expression 14-fold, 14-fold and 41-fold lower during infection with YFV, DENV and WNV, respectively. This indicates that the mosquito Jak-STAT pathway is likely involved throughout infection with all three flaviviruses. This also suggests that YFV, DENV and WNV may have evolved a conserved mechanism to suppress this antiviral pathway during infection. The Toll pathway has also been previously implicated in anti-flaviviral defense by the mosquito [10]. One gene shown to be involved in the mosquito Toll pathway and downregulated by DENV, AAEL001929, was 2.5-fold lower on D1 of infection with YFV and 2.5-fold lower on D2 of DENV infection in our study. Another Toll gene, AAEL003507, was only significantly downregulated during YFV infection, with 2.3-fold and 2.6-fold lower expression on D1 and D7, respectively. Our analysis also found a serine protease gene (AAEL006568) to be downregulated during infection with all three flaviviruses. Previous studies show that some midgut serine proteases limit DENV-2 infection in *Aedes aegypti* [19]. The *Drosophila* homolog of this gene, Spatzle-processing enzyme (CG16705), is known to be a positive regulator of the Toll pathway [35]. This implies that serine proteases may contribute to the innate immune response to viruses in mosquitoes.

We also demonstrate that virally-regulated gene expression is tissue-specific. Since flaviviruses must travel from the site of entry and infection, the midgut, throughout the mosquito body before reaching the salivary glands, it was likely that the expression of many genes would be differentially altered in various organs at different timepoints. Genes which are highly upregulated early in infection are likely important for flaviviral colonization of the midgut as well as the start of dissemination out of the midgut. Alternatively, these genes could represent the innate immune response of the mosquito to viral replication in the midgut. We saw several of the flavivirally-down regulated genes in the whole mosquito also downregulated by WNV infection in the midgut on D1 of infection, including AAEL011375, which was 17-fold lower than the mock group. This gene encodes a protein in the trypsin family, and trypsin silencing has been shown to increase DENV infection in *Aedes* [19]. One gene that was highly downregulated in the whole mosquito in response to infection with all three flaviviruses, AAEL003012, encodes the putative matrix metalloprotease (MMP) protein. This gene was also downregulated in the midgut, salivary gland and abdomen on D1 of WNV infection, suggesting that the protein may play a role in controlling the initial viral infection in the mosquito. Genes downregulated late in infection are possibly inhibitory to establishment of infection in the salivary glands or to transmission of virus to a new host. By D14 p.i, many of the identified virally-downregulated genes have lower expression in the salivary glands, which might be expected as the virus should be concentrated in this organ by this timepoint. Interestingly, this is the only organ in which the MMP protein is still downregulated at D14 p.i., which again indicates that this protein may be involved in controlling viral replication and/or infection.

Bioinformatics analysis revealed significant changes in the expression of genes from diverse cellular processes, including ion binding, ion transport, metabolic processes and peptidase activity. In a previous study investigating gene expression in midguts of WNV-infected *Culex* mosquitoes on D10 p.i., almost 5% of the highly upregulated genes were related to ion transport. This alteration in ion transport molecules was hypothesized to aid in viral spread through polarized cells by maintaining proper cell polarity and stable solute transport functions [12]. A study on the infection of Aedes with Sindbis virus (SINV) found genes involved in ion transport upregulated D4 p.i. [25]. The same group also saw a decrease in genes related to chitin binding on D1 of SINV infection and our analysis revealed a significant reduction in genes involved with chitin binding and the structural constituent of cuticle. Metabolism and oxidoreductive processes were previously found to be major functional groups altered by DENV infection in *Aedes* on D10 p.i. [10]. In agreement, our analysis showed that genes involved with oxidation reduction and zinc ion binding were highly upregulated and metabolic process and chitin metabolic process were highly downregulated. We also found that genes encoding serine protease inhibitors were significantly downregulated by infection with all three flaviviruses. In a study investigating mammalian genes important in WNV, it was found that silencing serine peptidase inhibitors significantly increased infection, indicating that a reduction in these proteins favors viral infection [36]. This highlights the likely overlap between mosquito and mammalian flaviviral host factors. We also looked at the cellular location of the DEGs found during infection. In our analysis, the majority of virally-upregulated genes produce intracellular and nuclear proteins and most of the virally-downregulated genes encoded proteins found in the extracellular region.

Several of the proteins encoded by the most significantly virally-downregulated genes are shown to be inhibitory to WNV infection both in mosquito cells and in mosquitoes *in vivo*. This is direct evidence that viral infection in the mosquito decreased the expression of proteins that are likely to impede viral replication or infection of new cells. Two of these proteins, a pupal cuticle protein and a matrix metalloprotease, were shown to alter the protein profiles of WNV E and NS1. In addition, the PC protein was able to directly bind WNV E protein and inhibit viral infection in mosquito cells as early as 3 hours post-infection. This suggests that PC is acting at the step of viral entry, likely by directly binding the E protein on the virus surface. The MMP protein was also able to inhibit WNV infection in cells, though at a later timepoint than PC. When mixed with live WNV, PC protein enhanced the survival of injected mice, indicating direct action on the virus to impede infection. These results provide strong evidence that the virally-downregulated genes identified in this study likely represent proteins that are inhibitory to flaviviral infection. To further ensure that our findings are relevant for WNV infection in nature, we aligned the pupal cuticle protein from *Ae. aegypti* with the corresponding *Cx. quinquefasciatus* protein and found 92% sequence identity. In addition, we performed a BLAST of the *Ae. aegypti* DDRGs that we use in the WNV studies against the *Cx. quinquefasciatus* genome and found very high sequence identity (85–95%). This indicates that the genes identified as inhibitory to WNV infection in *Aedes* mosquitoes are likely also inhibitory to infection in *Culex* mosquitoes.

This investigation uncovered many previously unknown host factors differentially regulated by flaviviral infection. This is also the first study, to our knowledge, to compare infection with three flaviviruses in the same mosquito at the same timepoints. Understanding the effects of infection on the mosquito, both common and unique to individual flaviviruses, will aid in developing broadly applicable methods to treat and prevent infection.

## Materials and Methods

### Cell culture and virus growth

The CCL-125 *Aedes aegypti* cell line (ATCC, VA) was used for transfection and infection studies. The cells were grown at 30°C and 5% CO2 in DMEM supplemented with 10% heat-inactivated fetal bovine serum (Gemini, CA), 1% penicillin-streptomycin and 1% tryptose phosphate broth (Sigma, MO). Flavivirus was grown in C6/36 *Aedes albopictus* cell line using the same media. Strains used were: WNV 2741, DENV-2 New Guinea C, YFV Asibi strain. Cells were infected at an m.o.i. of 1.0, virus was allowed to propagate for 6–8 days, supernatant was removed, spun down and virus stock was stored at −80°C until use.

### Mosquito infections

The Rockefeller strain of *Ae. aegypti* mosquitoes were either infected by intra-thoracic inoculation or blood-feeding, as indicated in the text and figure legends. For thoracic injections, virus was used at 6.5 logs per mL and 0.5 µL were injected per mosquito. For blood feeding, 100 µL of virus was added to 1 mL serum-inactivated blood from c57L/B6 mice and fed to mosquitoes for 20 minutes at room temperature using a hemotek feeder. Mosquitoes were maintained in groups of 10 at 30°C, 80% humidity. Mosquitoes were supplied raisins as a source of dietary sugar.

### Microarray analysis

RNA was isolated from WNV, DENV type 2 or YFV infected *Ae. aegypti* mosquitoes on days 1, 2 and 7. RNA was purified using the Rneasy kit (Qiagen, CA) and hybridized with Nimblegen X4 microarray chips using 81-mer probes designed from 18,000 open reading frames (ORF) found in the *Ae. aegypti* genome, with 2 different probes per ORF. The gene expression data was normalized using quantile normalization [37]. Partek Genomic Suite v. 6.4 (http://www.partek.com) was used for the statistical data analysis and ANOVA was applied to identify differentially expressed genes between each infection versus mock for each timepoint. False discovery rate was used to adjust the p-value for multiple testing corrections [38].

### Bioinformatics analysis

The functional annotation and clustering of DEGs was performed using the DAVID Bioinformatics Resource 6.7 [39], [40]. Briefly, IDs of DDRGs and DURGs were uploaded separately to the DAVID web interface and converted to unique DAVID IDs. The background gene list for the functional clustering consisted of the IDs of all *Ae. Aegypti* transcripts represented on the Nimblegen X4 microarray chips. The functional annotation and clustering were then performed using the DAVID default parameters.

### qRT-PCR analysis

RNA was isolated from infected Ae. aegypti mosquitoes on days 1, 2, 7 and 14 and purified using RNeasy kit (Qiagen, CA) according to manufacturer's instructions. cDNA was made from the RNA using a SuperscriptIII kit (Invitrogen, CA). cDNA from 1 µg RNA was used in each quantitative (q)RT-PCR reaction along with SYBR green chemistry. Fold change in gene expression was calculated using the CT value differences normalized to actin expression. Oligos can be found in Table S4.

### Immunofluorescence analysis

CCL-125 *Aedes aegypti* cells were infected with WNV at an MOI of 0.1. 24 hours post-infection, cells were fixed in 4% paraformaldehyde for 20 min at RT, washed with PBS(-) and then stained for infection using an antibody against recombinant WNV E protein conjugated with TRITC. The antibody was diluted in 1% BSA at 1/250 and cells were incubated for 20 minutes at RT. Infection was visualized using fluorescent microscopy.

### Protein production

GST-tagged protein was made from two mosquito genes: AAEL011045, which codes for a pupal cuticle protein, and AAEL003012, which produces a putative matrix metalloprotease. Protein was produced in *E.coli* and batch purified using glutathione sepharose (GS) (GE, NJ) with centrifugation. Briefly, pelleted bacteria from 1L culture were lysed and mixed with 2 mL GS resin with end-over-end mixing for 1 h at RT. The resin was spun down at 500 rpm, washed with PBS(-) and protein was eluted with buffer (50 mM Tris-HCl, 10 mM reduced glutathione, pH 8.0). The GST tags were removed using PreScission Protease enzyme (GE, NJ).

### ELISA analysis

Binding between the *Ae. aegypti* PC or MMP proteins with flaviviral proteins was investigated using ELISA analysis. Briefly, 5 µg of mosquito or GST control protein was coated onto a 96-well ELISA plate (Thermo Fisher Sci, MA) and incubated overnight at 4°C. The plate was blocked with 1% BSA in PBS(-) and incubated with 1 µg of flaviviral protein (either WNV E, WNV NS1, DENV E or DENV C) or BSA control for an hour at RT. The proteins were washed off, secondary-HRP was added for 30 min at RT, washed off and TMB substrate was added for 20 min at RT. Stop solution was added and the O.D. of the wells read at 450 nm. WNV NS1 protein was a kind gift from Dr. Michael Diamond (Washington University, MO), WNV and DENV E were kind gifts from L^2^ (CT) and recombinant DENV C was produced in our laboratory.

### Western blots

The *Ae. aegypti* PC or MMP proteins were incubated with each of four viral proteins (WNV E, WNV NS1, DENV E, DENV C) for one hour at 37°C. The solution was run on a 4-12% SDS-PAGE gel for 1.5 h at 15 milliamps per gel. The proteins were then transferred to nitrocellulose membrane. The nitrocellulose was blocked with 5% milk in 1% TBST for 1 h at RT and then incubated with the appropriate primary antibody overnight at 4°C. The nitrocellulose was washed and then incubated with the appropriate horseradish peroxidase secondary antibody for 1 h at RT. The protein blots were incubated with ECL substrates (Amersham, NJ) for 5 min at RT and then detected on Kodak film. Antibodies used: anti-WNV envelope (L^2^, CT), anti-dengue envelope (L^2^, CT), anti-WNV NS1 (gift from Dr. Michael Diamond, Washington University School of Medicine, MO) and mouse immune serum against recombinant DENV-2 capsid protein made in our laboratory.

### Mice

Nine week old female C57BL/6 mice were infected with WNV (with and without mosquito proteins) intraperitoneally (i.p.) at a dose of 10^3^ plaque forming units (pfu) per mouse. All animal experimental protocols were approved by the Institutional Animal Care and Use Committee of Yale University and experiments were done in a Biosafety Level 3 animal facility according to the regulations of Yale University.

### Transfection of plasmids

All plasmids were transfected into CCL-125 cells using Effectene (Qiagen, CA) according to manufacturer's instructions. Briefly, for a 10 cm^2^ plate, 10 µg of DNA was mixed with 500 µL buffer EC and 32 µL enhancer was added. This was allowed to incubate for 5 min on the benchtop. Then, 30 µL Effectene reagent was added and the solution vortexed briefly. After 10 min incubation, the solution was added to the cells. Expression was observed 24 h post-transfection and peaked at 48 h.

### Accession numbers

The following GENBANK accession numbers were referenced in the manuscript text: AAEL014440, AAEL004861-RA, AAEL011045, AAEL003012, AAEL012402, AAEL014108, AAEL004897, AAEL012770, AAEL000340, AAEL011375, AAEL009577, AAEL001704, AAEL001022, AAEL003685, AAEL009750, AAEL010160, AAEL001953, AAEL000611, AAEL000627, AAEL001929, AAEL003507, AAEL006568, AAEL011375.

### Ethics statement

Our study was carried out in strict accordance with the recommendations Guide for the Care and Use of Laboratory Animals of the National Institutes of Health. All animal experimental protocols were approved by the Institutional Animal Care and Use Committee of Yale University (Protocol Permit Number: 2008-07941) and experiments were done in a Biosafety Level 3 animal facility according to the regulations of Yale University. All efforts were made to minimize suffering.

## Supporting Information

Figure S1 **Summary of the microarray data.** *Ae. aegypti* mosquitoes were infected with WNV, DENV or YFV and microarray analysis was done using RNA isolated on days 1, 2 and 7 post-infection. Mosquito genes that were ≥5-fold up-regulated (203 genes) and ≥10-fold down-regulated (202 genes) during infection with any virus at any timepoint are designated differentially expressed genes (DEGs). A. Graph plots the trend of the DEGs from D0-D8 for all 3 FVs, blue line indicates positive regulation, red line indicates negative regulation. B. Chart showing the number of DEGs up- or down-regulated for each FV at each timepoint.
(PDF)

Figure S2 **Detailed heatmaps for DEGs from microarray analysis.** A–G. Detailed heatmaps for *Ae. aegypti* genes that were ≥5-fold up-regulated (203 genes) and ≥10-fold down-regulated (202 genes) during infection with any virus at any timepoint. Flavivirus and timepoint are indicated at the bottom of each heatmap, individual genes are listed on the right.
(PDF)

Figure S3 **Immunofluorescence analysis of WNV-infected** ***Ae. aegypti*** **cells.** CCL-125 cells were infected with WNV at an MOI of 0.1 and fixed with 4% paraformaldehyde 24 hours p.i. Cells were stained with an antibody against the WNV envelope protein conjugated to TRITC secondary. Scale bar is shown in lower right corner.
(PDF)

Figure S4 **Whole body transfection of** ***Ae. aegypti*** **mosquitoes.** Mosquitoes were injected via intra-thoracic inoculation with insect expression vectors coding for AAEL003012 (MMP) or AAEL011045 (PC). RNA was isolated from mosquitoes on day 3 and day 14 post-transfection and qRT-PCR analysis was done to detect levels of expression. Mosquitoes that received the plasmid coding for the alternate gene were used as controls for expression of each gene. Each point represents 10 mosquitoes; fold increase in expression is indicated.
(PDF)

Figure S5 **Mosquito MMP and PC change the protein profile of WNV proteins.** Recombinant MMP protein (AAEL003012) was incubated with WNV NS1 (A), WNV E (B) and PC protein (AAEL011045) was incubated with WNV E (C) for an hour at 37°C. Solutions were run on a 12% SDS-PAGE gel. The proteins were transferred to nitrocellulose and Western blot analysis was done using antibodies against the viral proteins. 5 mM ZnCl was added to one solution of MMP and viral proteins as MMP is a presumed metalloprotease. Dimers and monomers are indicated.
(PDF)

Table S1 **Microarray analysis data.** *Ae. aegypti* mosquitoes were infected with WNV, DENV or YFV and microarray analysis was done using RNA isolated on days 1, 2 and 7 post-infection. To identify fold change in expression, we applied ANOVA to each infected sample versus mock for each timepoint and applied false discovery rate (FDR) to adjust the p-value for multiple corrections. Submitted to NCBI Gene Expression Omnibus.
(XLS)

Table S2 **Functional annotations of DEGs.** DURGs and DDRGs were assigned to functional categories for biological process, molecular function and cellular component.
(XLS)

Table S3 **Functional clustering of DEGs.** DURGs and DDRGs were assigned to functional categories for biological process, molecular function and cellular component. Genes were analyzed for functional clustering. Enrichment scores are listed for each cluster.
(XLS)

Table S4 **Oligos used for qRT-PCR.** qRT-PCR was done with listed oligos to determine levels of gene expression, normalized to actin expression.
(XLS)
