# Supplementary figures and images for: Alterations in the Aedes aegypti Transcriptome during Infection with West Nile, Dengue and Yellow Fever Viruses
Source: PLoS Pathog. 2011 Sep 1;7(9):e1002189. doi: 10.1371/journal.ppat.1002189 (PMC3164632; doi:10.1371/journal.ppat.1002189)

A

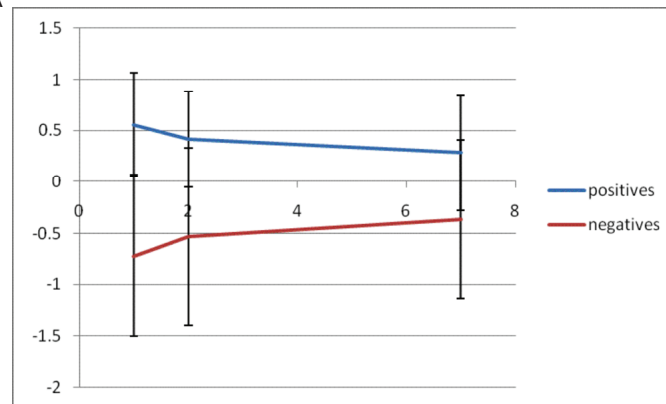

B

|       | Day1 |      | Day 2 |      | Day 7 |      | Total |      |
|-------|------|------|-------|------|-------|------|-------|------|
|       | Up   | Down | Up    | Down | Up    | Down | Up    | Down |
| DENV  | 53   | 97   | 13    | 74   | 9     | 15   | 65    | 81   |
| WNV   | 33   | 28   | 24    | 11   | 10    | 7    | 59    | 40   |
| YFV   | 82   | 63   | 28    | 22   | 90    | 21   | 164   | 84   |
| Total | 168  | 188  | 65    | 107  | 109   | 43   | 288   | 205  |

Supplement: Figure S1 — Summary of the microarray data. Ae. aegypti mosquitoes were infected with WNV, DENV or YFV and microarray analysis was done using RNA isolated on days 1, 2 and 7 post-infection. Mosquito genes that were ≥5-fold up-regulated (203 genes) and ≥10-fold down-regulated (202 genes) during infection with any virus at any timepoint are designated differentially expressed genes (DEGs). A. Graph plots the trend of the DEGs from D0-D8 for all 3 FVs, blue line indicates positive regulation, red line indicates negative regulation. B. Chart showing the number of DEGs up- or down-regulated for each FV at each timepoint. (PDF) [file ppat.1002189.s001.pdf]

A

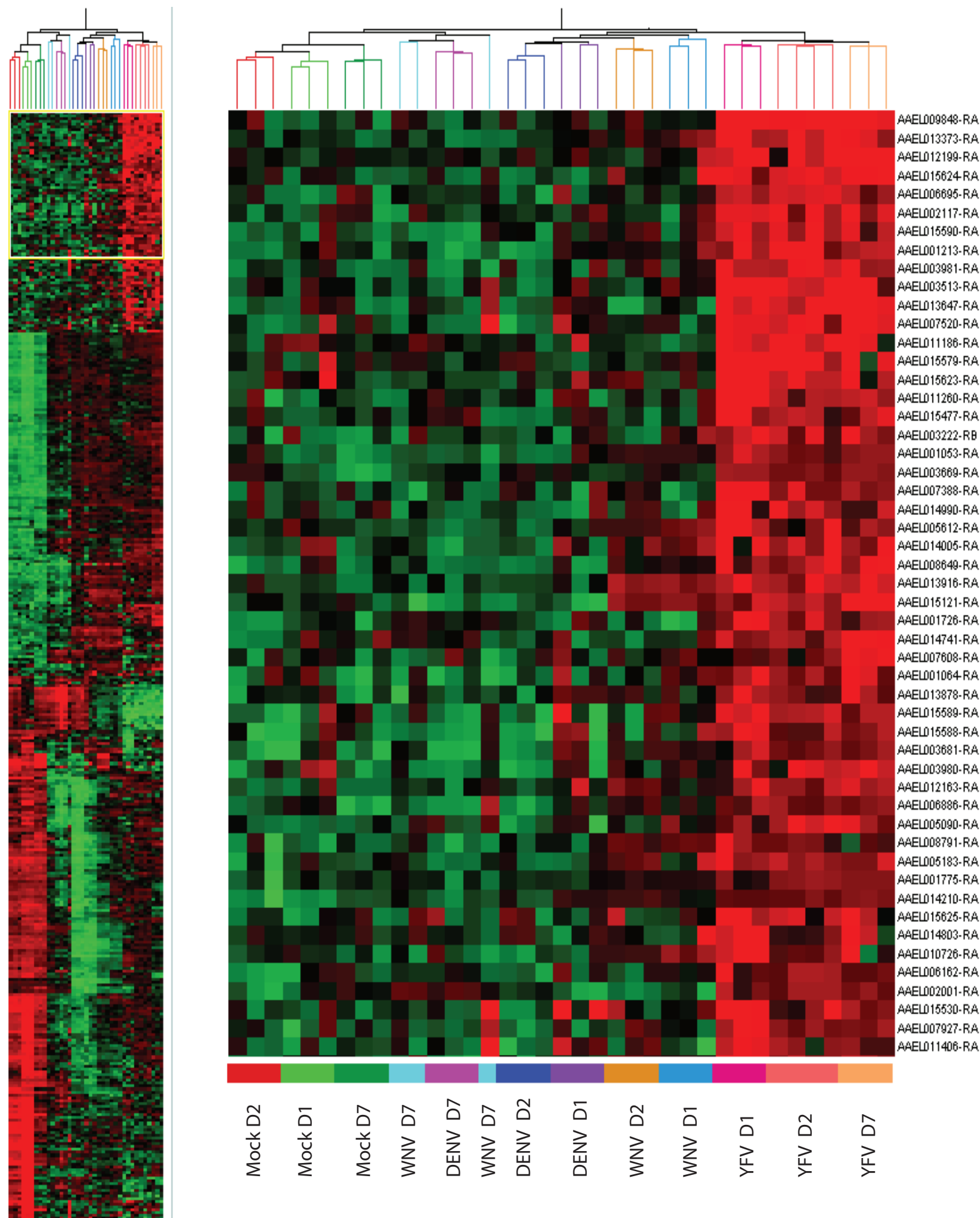

B

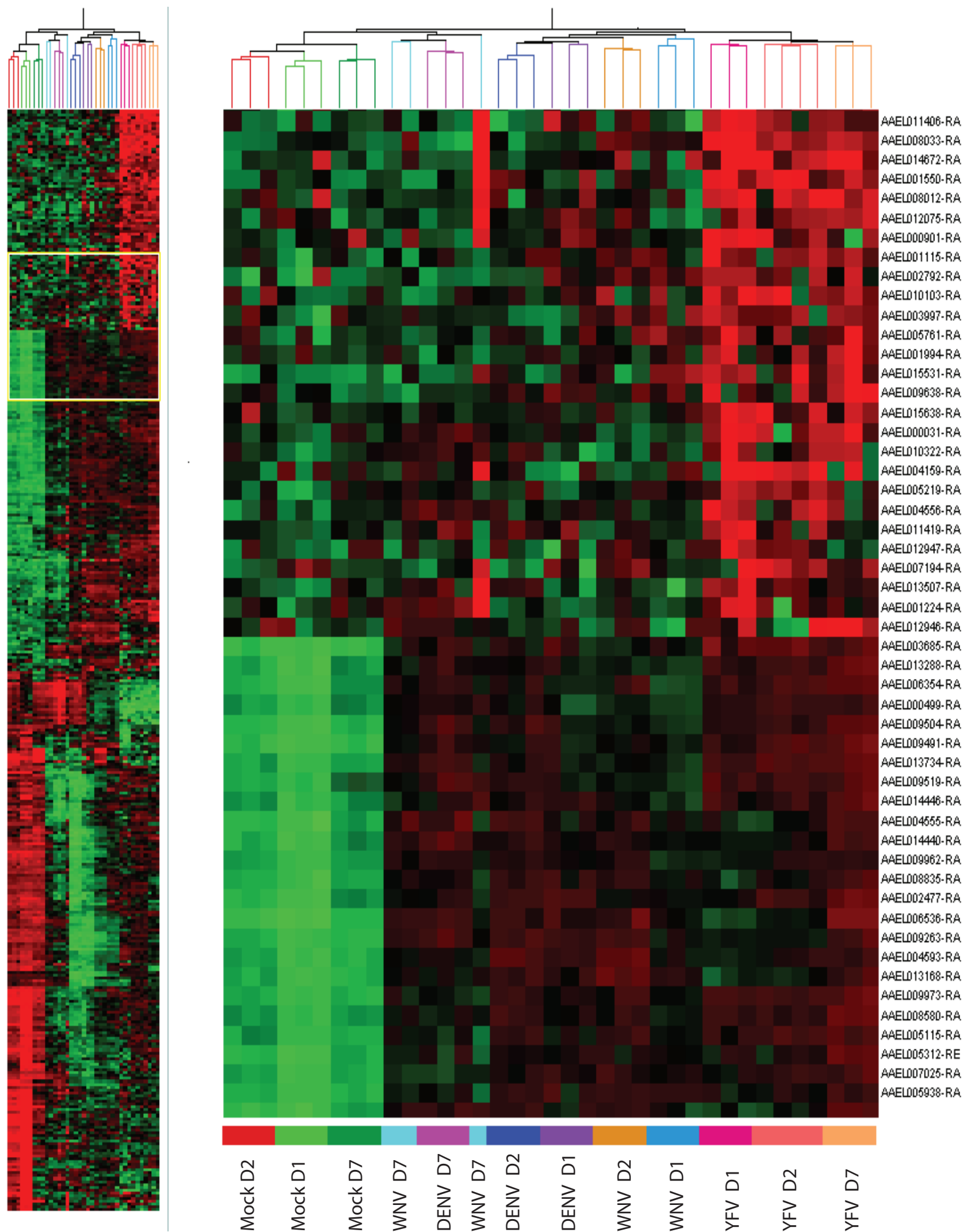

C

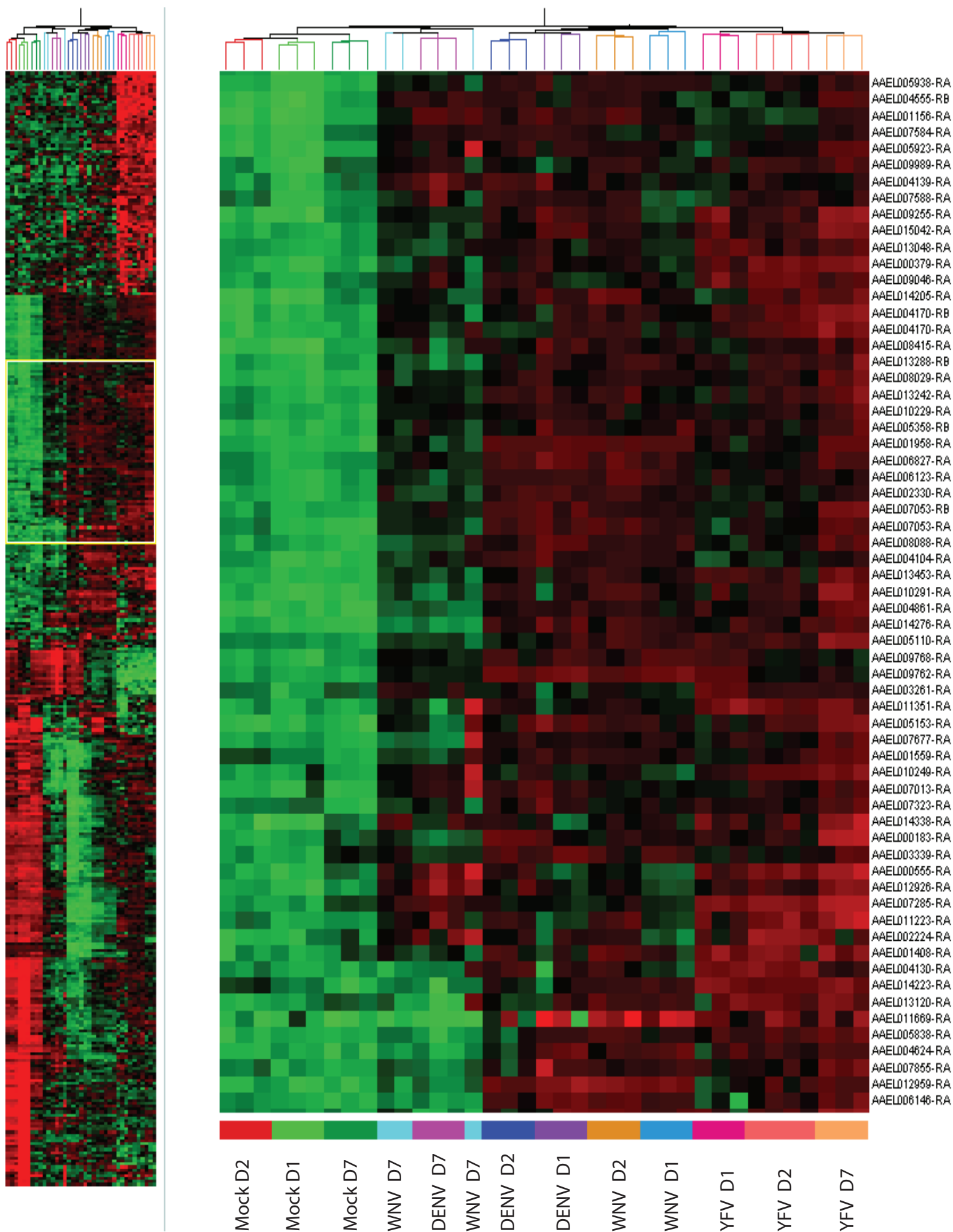

D

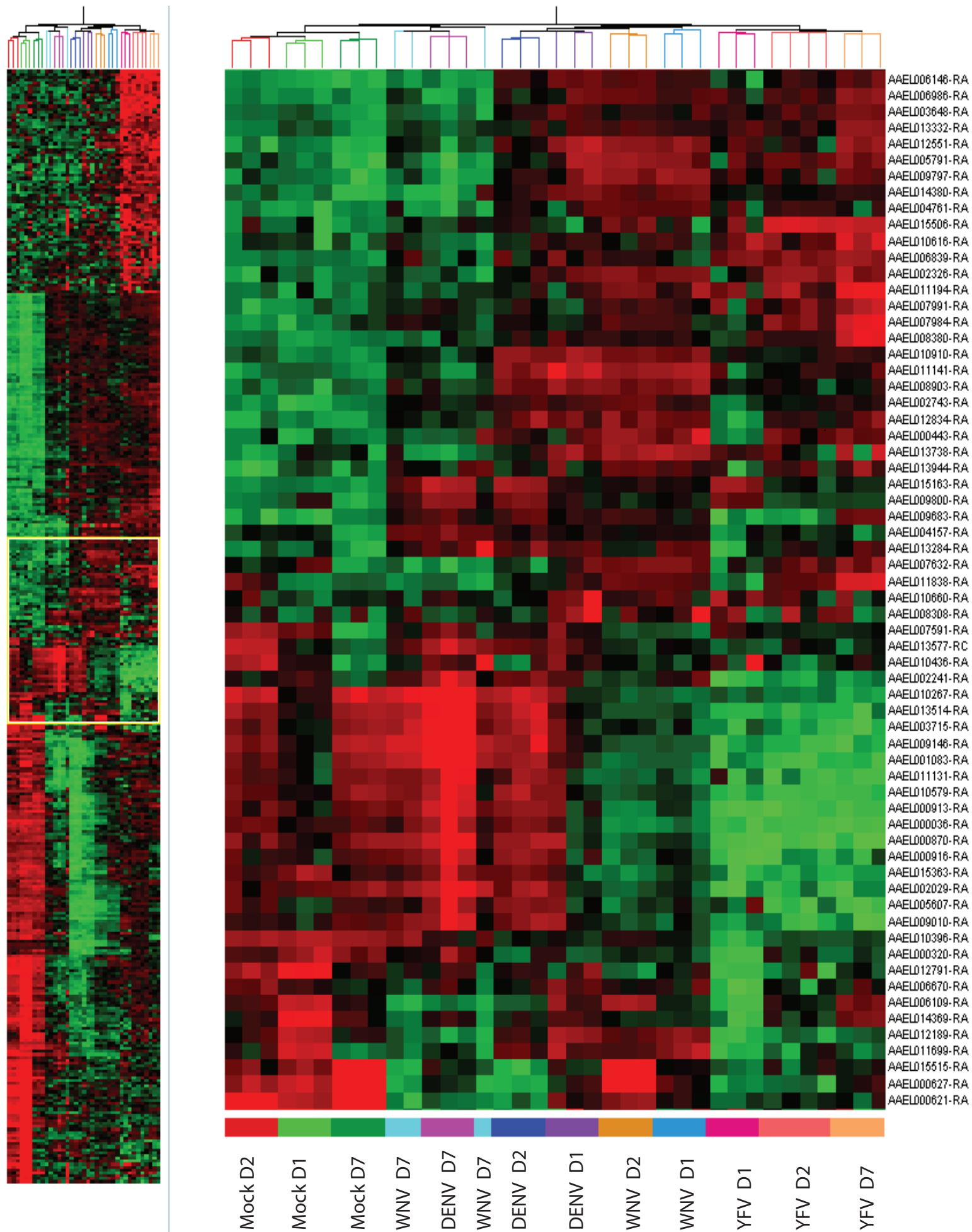

E

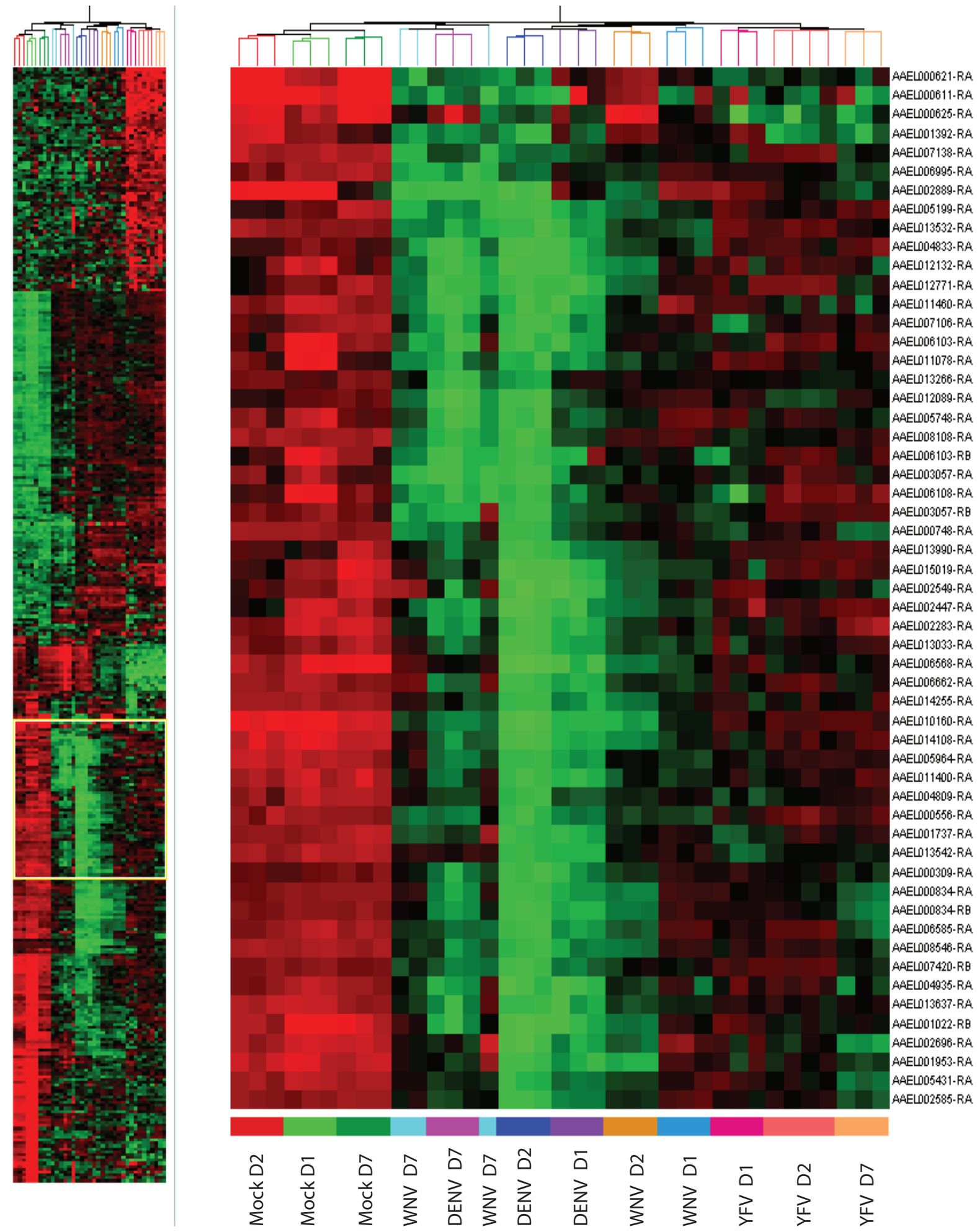

F

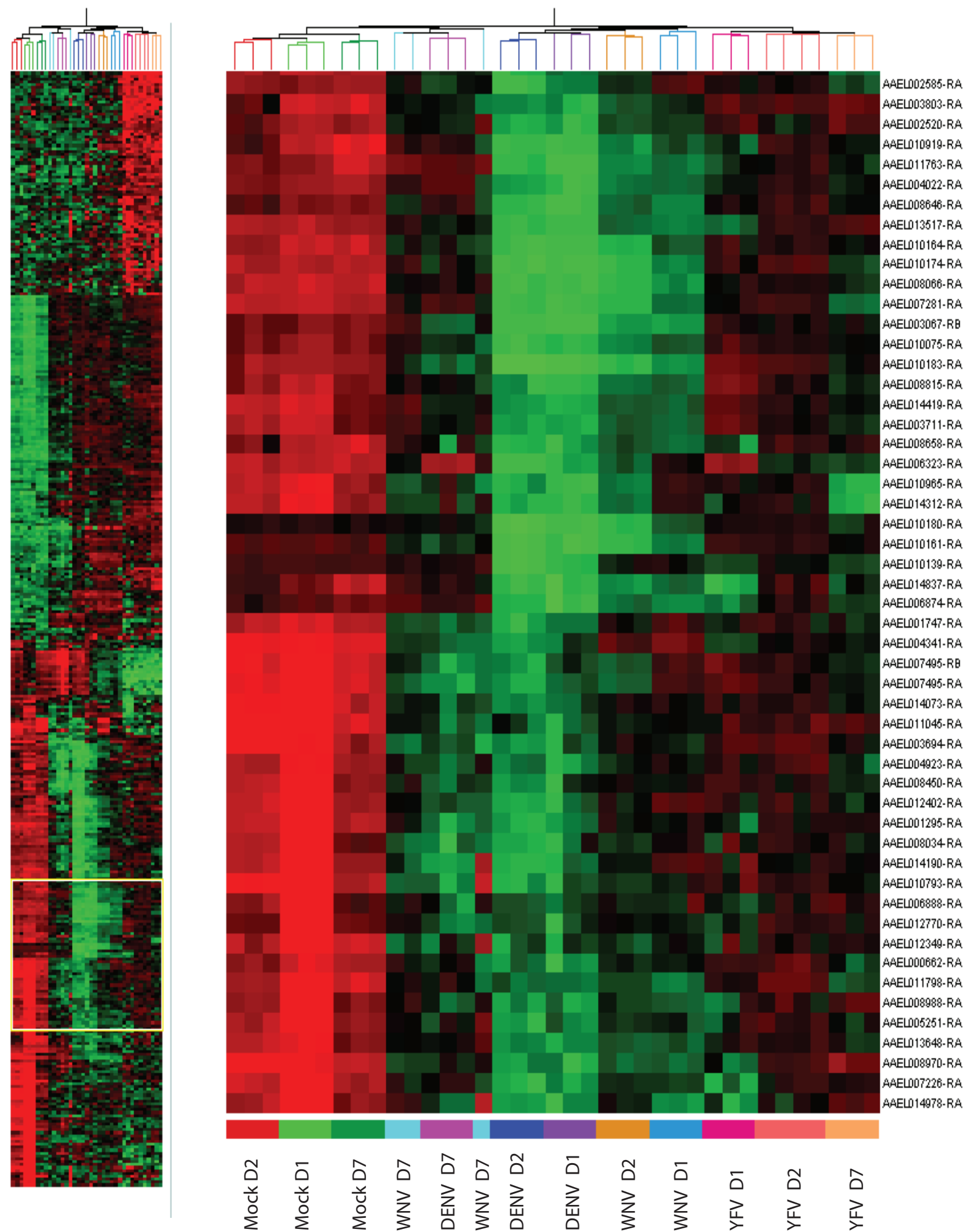

G

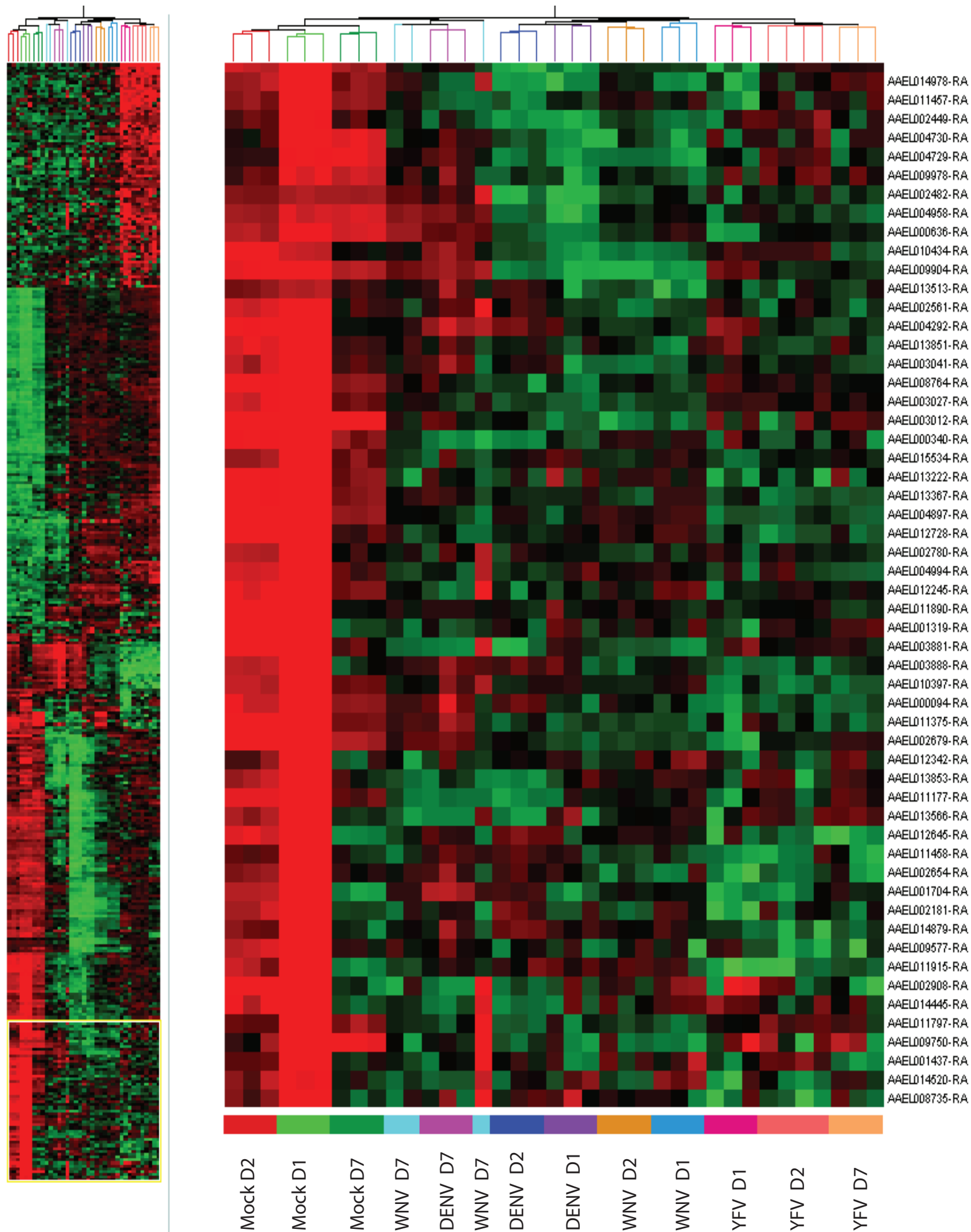

Supplement: Figure S2 — Detailed heatmaps for DEGs from microarray analysis. A–G. Detailed heatmaps for Ae. aegypti genes that were ≥5-fold up-regulated (203 genes) and ≥10-fold down-regulated (202 genes) during infection with any virus at any timepoint. Flavivirus and timepoint are indicated at the bottom of each heatmap, individual genes are listed on the right. (PDF) [file ppat.1002189.s002.pdf]

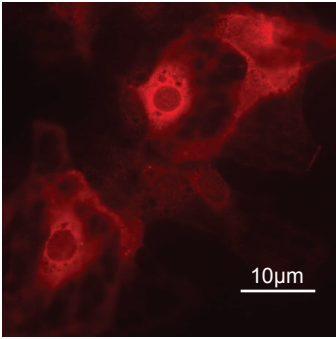

Supplement: Figure S3 — Immunofluorescence analysis of WNV-infected Ae. aegypti cells. CCL-125 cells were infected with WNV at an MOI of 0.1 and fixed with 4% paraformaldehyde 24 hours p.i. Cells were stained with an antibody against the WNV envelope protein conjugated to TRITC secondary. Scale bar is shown in lower right corner. (PDF) [file ppat.1002189.s003.pdf]

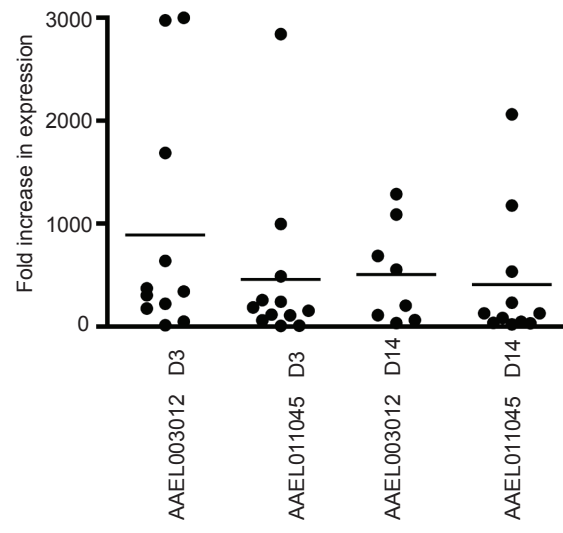

Supplement: Figure S4 — Whole body transfection of Ae. aegypti mosquitoes. Mosquitoes were injected via intra-thoracic inoculation with insect expression vectors coding for AAEL003012 (MMP) or AAEL011045 (PC). RNA was isolated from mosquitoes on day 3 and day 14 post-transfection and qRT-PCR analysis was done to detect levels of expression. Mosquitoes that received the plasmid coding for the alternate gene were used as controls for expression of each gene. Each point represents 10 mosquitoes; fold increase in expression is indicated. (PDF) [file ppat.1002189.s004.pdf]

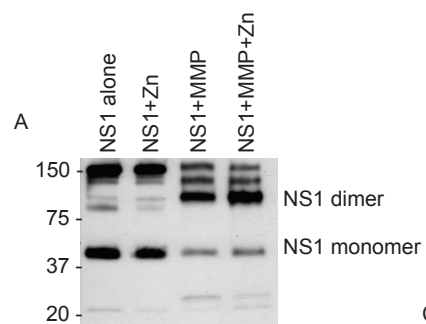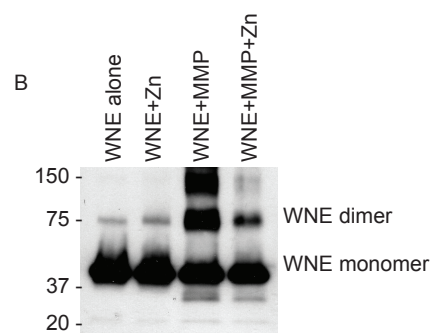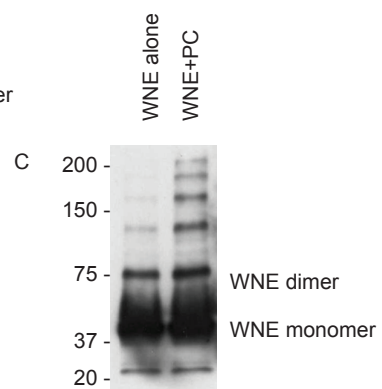

Supplement: Figure S5 — Mosquito MMP and PC change the protein profile of WNV proteins. Recombinant MMP protein (AAEL003012) was incubated with WNV NS1 (A), WNV E (B) and PC protein (AAEL011045) was incubated with WNV E (C) for an hour at 37°C. Solutions were run on a 12% SDS-PAGE gel. The proteins were transferred to nitrocellulose and Western blot analysis was done using antibodies against the viral proteins. 5 mM ZnCl was added to one solution of MMP and viral proteins as MMP is a presumed metalloprotease. Dimers and monomers are indicated. (PDF) [file ppat.1002189.s005.pdf]
